# Developing the Philippines as a Global Hub for Disaster Risk Reduction - A Health Research Initiative as Presented at the 10th Philippine National Health Research System Week Celebration

**DOI:** 10.1371/currents.dis.5cf90566bb7791456dcf6b9baf6d4873

**Published:** 2016-10-25

**Authors:** Nicola Banwell, Jaime Montoya, Merlita Opeña, Carel IJsselmuiden, Ronald Law, Gloria J. Balboa, Shannon Rutherford, Cordia Chu, Virginia Murray

**Affiliations:** Centre for Environment and Population Health, Griffith University, Brisbane, Queensland, Australia; Department of Health, Manila, Philippines; Philippine Council for Health Research and Development, Department of Science and Technology, Manila, Philippines; Philippine Council for Health Research and Development, Department of Science and Technology, Manila, Philippines; Council on Health Research for Development (COHRED), Geneva, Switzerland; School of Applied Human Sciences, University of KwaZulu-Natal, Durban, South Africa; Health Emergency Management Bureau, Department of Health, Manila, Philippines; Health Emergency Management Bureau, Department of Health, Manila, Philippines; Centre for Environment and Population Health, Griffith University, Brisbane, Queensland, Australia; Centre for Environment and Population Health, Griffith University, Brisbane, Queensland, Australia; Public Health England, London, England; UNISDR Scientific and Technical Advisory Group, Geneva, Switzerland; Integrated Research on Disaster Risk Scientific Committee, Beijing, China

## Abstract

The recent Philippine National Health Research System (PNHRS) Week Celebration highlighted the growing commitment to Disaster Risk Reduction (DRR) in the Philippines. The event was lead by the Philippine Council for Health Research and Development of the Department of Science and Technology and the Department of Health, and saw the participation of national and international experts in DRR, and numerous research consortia from all over the Philippines. With a central focus on the Sendai Framework for Disaster Risk Reduction, the DRR related events recognised the significant disaster risks faced in the Philippines. They also illustrated the Philippine strengths and experience in DRR. Key innovations in science and technology showcased at the conference include the web-base hazard mapping applications ‘Project NOAH’ and ‘FaultFinder’. Other notable innovations include ‘Surveillance in Post Extreme Emergencies and Disasters’ (SPEED) which monitors potential outbreaks through a syndromic reporting system. Three areas noted for further development in DRR science and technology included: integrated national hazard assessment, strengthened collaboration, and improved documentation. Finally, the event saw the proposal to develop the Philippines into a global hub for DRR. The combination of the risk profile of the Philippines, established national structures and experience in DRR, as well as scientific and technological innovation in this field are potential factors that could position the Philippines as a future global leader in DRR. The purpose of this article is to formally document the key messages of the DRR-related events of the PNHRS Week Celebration.

## Introduction

The 10th annual Philippine National Health Research System (PNHRS) Week Celebration, from the 8th to 12th August 2016 in Puerto Princesa, Palawan, Philippines, brought together health researchers, policy makers and practitioners from across the Philippines. The conference theme Research and Innovation for Health and the Environment aimed to facilitate smoother exchange of health-related research among key stakeholders via ten pre-conference events, seven parallel sessions, and two plenary sessions. This year’s conference saw an unprecedented focus on Disaster Risk Reduction (DRR). The purpose of this publication is to formally document and communicate the key messages that emerged from the conference.

The 10th PNHRS Week Celebration placed the Sendai Framework for Disaster Risk Reduction (Sendai Framework) at the front and centre of its agenda, highlighting the growing recognition among researchers and policy makers of the need to bolster research in this area within the Philippines. The key DRR-focused events included the Pre-forum workshop on Framework for Disaster Research in Health, the parallel session National Health Research Program on Disaster Risk Reduction and the plenary session on The Philippines as a Research Hub on Global Health Innovations to Deal with Climate Change and Natural Disasters. These events highlighted key strengths and challenges of DRR research in the Philippines within the broader context of positioning the Philippines as a global hub for research and innovation in DRR.

The year 2015 has been noted as a historic year in international policy, with the finalisation of three landmark United Nations agreements. These are:

- The **Sendai Framework** that focuses on reducing disaster risks and losses in terms of lives, livelihoods and health^1^^,^ 2 . It was adopted in Sendai, Japan in March 2015 by 187 member states and endorsed by the UN General Assembly in June 2015^3^ .
- The **Sustainable Development Goals** (SDGs), which followed on from the Millennium Development Goals, and saw the significant positioning of health within a specific health goal with notable links to other goals of the SDGs^4^. The SDGs were agreed in New York, USA by 193 countries^3^.
- The **Paris Agreement on Climate Change **was finalized and agreed upon by 195 countries in December at the Paris Climate Conference (CoP21)^3^.

Further to these agreements, the Sendai Framework was followed by the UNISDR Science and Technology Conference on the Implementation of the Sendai Framework for Disaster Risk Reduction 2015–2030 in January 2016 which included in its recommendations the ‘Need for formal ‘‘national DRR science-policy councils/platforms’’ or a form of national focal points for science to support disaster risk reduction and management plans identified’ 3. A second conference on the implementation of the health aspects of the Sendai Framework for Disaster Risk Reduction 2015-2030^5^ in March 2016 lead to the Bangkok Principles which aim to strengthen health implementation of DRR^5^. Additionally, the landmark paper of the 2015 Lancet Commission on Health and Climate Change, called for health to play a larger role in tackling climate change, viewing climate change as an opportunity and necessary area of strengthened action for the health sector in coming decades 6.

## Background to the impact of disasters on the Philippines

Widely recognised as one of the most disaster-prone countries in the Asian Region, and the world 7, the Philippines was ranked second on the World Risk Index in 2014 in terms of exposure and risks to natural hazards 8. The Philippines was also the 5th most affected country by natural hazards from 1994 to 2013, and ranked as the most affected country in 2013 according to the 2015 Global Risk Index^9^. Between 1993 and 2012, the Philippines experienced 311 extreme weather events, the highest number globally, and falls within the top ten countries in the world most affected by extreme weather^7^. Most recently, the Philippines was found to have the highest expected annual mortality, affected population, and loss in GDP globally in relation to climatic hazards 10. This data is to the exclusion of impacts associated with non-climatic, biological or technological hazards.

The country is exposed to a variety of hazards across all categories – natural, biological, technological and social hazards such as mass gatherings^11^. Several geographic factors contribute to the high natural hazard exposure of the Philippines, including the country’s location in the ‘Pacific Ring of Fire’ at the junction of two large tectonic plates, the Philippine Sea Pacific Plates and the Eurasian Plate, facing the Pacific Ocean^12^ and one of the most active typhoon belts in the world 13.

In addition to these exposure factors, significant vulnerability as a result of inequity in access to healthcare and social protection mechanisms, as well as rapid unplanned urbanisation and development in economic hotspots, contribute greatly to the disaster risks faced by the population and economy of the Philippines 8 ^,^ 14.

DRR in the context of climate change has become a national priority with structures established to address these challenges. The national government has enacted the Climate Change Act of 2009 (RA 9729) and established the Climate Change Commission at the national level 15. The National Disaster Risk Reduction and Management Act of 2010 (DRRM Act, RA. 10121) has also been enacted and corresponding structures established 16. These structures are known as the National Disaster Risk Reduction and Management Council (NDRRMC), and are replicated at regional levels, known as Regional Disaster Risk Reduction and Management Councils (RDRRMC). National frameworks and plans in DRR and climate change have been developed and are in various stages of implementation 17 with the sunset review of the DRRM Act currently underway. Within the Department of Health (DOH), health emergency preparedness and response structures are institutionalised at the national level through the Health Emergency Management Bureau^18^.

The combination of these three factors, the risk profile, experiences and established structures in DRR, positions the Philippines to potentially become an international leader and global hub for DRR. However, several aspects need strengthening to support the development of the Philippines as an international hub for DRR. The key areas for strengthening identified through analysis of the content presented at the PNHRS Week Celebration include: integrated national hazard assessment, strengthened collaboration, and improved documentation.

## Science and technology innovations in the Philippines and areas for further development

In addition to the institutional structures mentioned above, the Philippines has relevant scientific and technical structures to contribute to the understanding of hazards and risks, and development of scientific innovation in DRR. These are coordinated by the Department of Science and Technology (DOST), and include, but are not limited to, the Philippine Institute of Volcanology and Seismology (PHIVOLCS) and the Philippine Atmospheric Geophysical and Astronomical Services Administration (PAGASA), to contribute to the understanding of hazards and risks, and development of scientific innovation in DRR.

Local governments are mandated to mainstream DRR and CCA in their local Comprehensive Land Use Plan (CLUP) and Comprehensive Development Plan (CDP). It is intended that these plans use vulnerability analysis and assessment within an integrated DRR and CCA framework 19 ^,^ 20 ^,^ 21. Further to this, Health Emergency Preparedness and Response and Recovery Plans at a regional and local government level contain natural hazard assessments for their corresponding areas 21. These hazard assessments provide potential sources for contributing to a detailed and integrated all-hazard assessment for the nation.

Examples of recent innovations in hazard mapping and assessment showcased during the PNHRS Week Celebration include the Nationwide Operational Assessment of Hazards, known as ‘Project NOAH’ and ‘FaultFinder’. Both of these web-based applications providing information on various hazards in the country, including meteorological and climatologically hazards, as well as major fault systems and earthquake risk mapping. Using a layered approach to mapping hazards, Project NOAH, allows users to select or search for a location and provides weather updates, data on rainfall and river inundation, as well as real time information on rain, weather and tides. The web-GIS tool provides hazard maps for floods, landslides and storm surge. It provides updates on flood reports, information on jurisdictions and critical infrastructure, as well as an impact assessment in the event of a hazard (available at: http://noah.dost.gov.ph/) 22. FaultFinder maps active fault systems, and allows users to search active fault systems of interest using GPS location on their mobile device, by name of location, and by browsing a detailed map view (available at: http://faultfinder.phivolcs.dost.gov.ph/) 23. These projects aim to advance scientific research and risk communication.

A health-based technological innovation cited by the Department of Health at the PNHRS Week Celebration was ‘Surveillance in Post Extreme Emergencies and Disasters’ (SPEED). Developed through a collaboration between the Philippines Department of Health and the WHO Philippines, SPEED uses web-based software to assist in gathering data relating to communicable and non-communicable diseases and conditions in extreme emergencies and disasters 24. SPEED gathers syndromic information from health facilities such as Evacuation Centers and Barangay Health Stations or Rural Health Units, and initial diagnoses from hospitals and private clinics 24. Data can be entered via manual encoding, SMS, or online. SPEED enables the monitoring of trends and early detection of disease outbreaks with the aim of providing timely and appropriate health response to minimise morbidity and mortality in an emergency or disaster 24.

Challenges in collaboration on DRR research in health was a key challenge noted by PNHRS conference participants. A key barrier to collaboration, which was noted by participants during the event, was the lack of awareness and documentation of DRR research and activities. Further to this, it was observed that there was limited representation of other government sectors, UN agencies, private sector and NGOs present at the event. Strengthened documentation of DRR activities, as well as involvement of these stakeholders in relevant future events will help to promote the establishment of the Philippines as a hub for DRR and build full cross-sectoral and cross-stakeholder engagement in the initiative.

Barriers to documentation of DRR research, policies and activities highlighted during the PNHRS Week Celebration include: lack of prioritisation of DRR research and documentation, as well as a lack of system and capacity for documentation. Prioritisation of DRR research is particularly absent in the context of health-related research, with the current National Unified Health Research Agenda for 2011 to 2016 making no direct mention of disaster-related research in health 25. While DRR research in health was acknowledged by panelists to be occurring, this research is happening on a limited scale, and is not included in existing national health research databases.

Two national registries for health–related research were promoted at the PNHRS Week Celebration, including the Health Research and Development Information Network (HERDIN) and the Philippine Health Research Registry. A recent search of these databases reveals limited documentation of DRR research in health and for those that were documented there was limited availability of related publications and outcome documents. At this point there exists no central system for documenting disaster-specific research. Key messages from the DRR sessions at the PNHRS Week Celebration included a need to prioritise and document DRR research if the country which could be developed as an output from possible global hub for DRR.

The conference participants considered that it would be beneficial to strengthen the documentation of DRR research and strategies to build the credibility and evidence base for the Philippines as an international exemplar for DRR and disaster risk management (DRM). To address the challenges in documentation, it would be helpful to consider the need for:

- capacity development with wider engagement through research mentoring;
- a system for documentation in each science and technology sector facilitating a centrally held inventory of DRR research across sectors and agencies;
- the improvement of capacity for peer-reviewed publication;
- improved library facilities for accessing peer review and grey literature resources;
- a facilitated rapid ethical clearance processes and the possible establishment of pre-disaster guidelines for research to be undertaken at the time of a disaster and in its aftermath.

## Proposal for the Philippines developing a Global Hub for DRR

Promotion of the Philippines as a global hub for DRR research and innovation to support the implementation of the Sendai Framework has the potential to strengthen DRR investment in the country. The establishment of relevant laws, structures, and technical capability demonstrates the importance of, and existing commitment to, DRR in the Philippines. These factors lend themselves well to this development of the Philippines as a global hub for DRR and DRM.

Experts and policy makers whom attended the PNHRS event frequently referred to the Philippines as a ‘laboratory’ of disasters in Asia. Panelists at the PNHRS identified the importance of climate change and the role of the health and the wider scientific community in developing the scientific and technological capacity of the Philippines in DRR. Within this context, panelists recognised not only the extensive risk profile of the country, but also the significant knowledge and experience developed in DRR and DRM through its established DRR structures.

The concept of developing a national focus in science and technology in the Philippines started more than two years ago as a key outcome of the partnership between the PCHRD and COHRED (the Council on Health Research for Development, accessible via this link: http://www.cohred.org/). Through this partnership the first Global Forum for Research and Innovation for Health in Manila in August 2015, “Forum 2015”, was hosted jointly by COHRED, PCHRD, DOH and DOST 26 ^,^ 27. The ‘hub’ concept intends to create a focus for national development, particularly through inter-departmental and inter-sectoral action, as well as international collaboration 28. Developing a ‘hub’ also aims to optimize the socio-economic impact of investments in science and technology.

Initial interest in the Philippines of becoming a leader in shaping the global research agenda in health and science more broadly was stated at Forum 2015. The event focused on how research and innovation can improve food security and nutrition, health in megacities and, most importantly, DRR. The DRR events that took place during Forum 2015, showcased examples in DRR from several nations, including the experience of the Philippines in strengthening and mobilizing local government units and communities for DRR 27. In the period following Forum 2015, COHRED and PCHRD outlined the field of concentration more sharply in a first concept paper prepared for DOST and DOH: the interface between science and innovation and the impact of disasters – or DRR – with health as a key outcome measure 28. This then became the basis for further internal and external consultations and for making this the focus of the 10th PNHRS Anniversary meeting in Palawan.

At the recent 10th Philippine National Health Research System (PNHRS) Week Celebration, the rationale for developing the Philippines as a hub for DRR was presented and well supported by the panelists from the PNHRS, the Department of Science and Technology, Department of Health and key national and international academic and private sector stakeholders present. During the plenary session, key stakeholders in DRR and health and the wider sciences and private sector demonstrated widespread support for the push to develop the Philippines as a global hub for innovations to deal with climate change and natural disasters, using an all hazard approach. Panelists in the plenary placed health as a central contributor to DRR, particularly recognizing that ‘zero casualty is not zero damage to health’ 29, and the need to reduce hazard exposure and vulnerability, as well as increase coping capacity within the context of health innovations for climate change and disasters 29. Panelists made note of the established structures and human resources that are already committed toward DRR in the Philippines, as well as the desire to share the experiences and expertise of the Philippines in addressing disaster risks 30. With the Sendai Framework providing a method to build research activities and outputs in order to enhance DRR capabilities, the Philippines could position itself as a global hub on DRR 31. However, there is a clear deficit in documentation and publication of these experiences and expertise, which needs to be addressed 32. Panelists also showcased the growing engagement of the private sector in strengthening DRR and clear support for developing the Philippines as a hub for DRR 33. Placing the Philippines at the centre of the converging points on health and the wider sciences addressing sustainable development, DRR and Climate Change Adaptation would be beneficial 34.

The positioning of the Philippines as a global hub could require significant financial investment, however, this has the potential to provide return on investment in DRR 35. An important reference was made to the triple dividend of DRR investment 36, where:

- the single dividend represents an approach that saves lives;
- the double dividend represents an approach that builds social and economic protection of livelihoods and assets including infrastructure and essential services; and
- the triple dividend represents economic co-benefits as a spinoff of technological innovation in DRR leading to a source of economic development for the country.

Overwhelming support by key political stakeholders was demonstrated for developing the Philippines into a global hub. This was echoed in the strong rationales presented by panelists in the DRR-related events, as well as by the attendees of the conference. The panelists of the DRR-related sessions clearly presented compelling reasons why the Philippines is well-positioned to become the global hub for DRR. These reasons include the disaster risk profile of the Philippines, experience in DRR and established structures necessary to support the initiative.

## Conclusion

As a consequence of its disaster incidence, especially with regards to the frequency and intensity of climate-related extreme events, the Philippines is widely recognised as one of the most at-risk countries in the world, with a developed strength and experience in DRR. This conference demonstrated the emergence of commitment towards using these experiences to strengthen DRR at Barangay, local, regional and national levels. The equal commitment demonstrated towards sharing these outputs with other at-risk countries globally is the driving force behind developing a global hub in the Philippines. The event also highlighted health research as a pivotal area for development in strengthening DRR in the Philippines towards the development of a global hub.

The commitment to the hub was announced at the 10th Philippine National Health Research System Week Celebration. Continuing work to determine what this might mean and how this concept might develop, particularly in the context of science and technology for health in DRR, will be undertaken prior to the presentation at the Global Platform for Disaster Risk Reduction to be held in May 2017 in Cancun, Mexico on 22-26 May. The Global Platform is the premiere international forum dedicated to the international DRR agenda and the 2017 Global Platform will be the first opportunity for initial assessments of the progress towards the Sendai Framework (The Global Platform website can be accessed via this link: http://www.unisdr.org/conferences/2017/globalplatform).

## Competing Interests

The authors would like to make it known that Virginia Murray is an editor on the Review Board for PLOS Currents: Disasters.

## Corresponding Author

Primary: Banwell, Nicola M.; Email: nicola.banwell@griffithuni.edu.au

Secondary: Opeña, Merlita M.; Email: mmopena@pchrd.dost.gov.ph
